# Supplementary figures and images for: Origin of circulating free DNA in patients with lung cancer
Source: PLoS One. 2020 Jul 7;15(7):e0235611. doi: 10.1371/journal.pone.0235611 (PMC7340299; doi:10.1371/journal.pone.0235611)

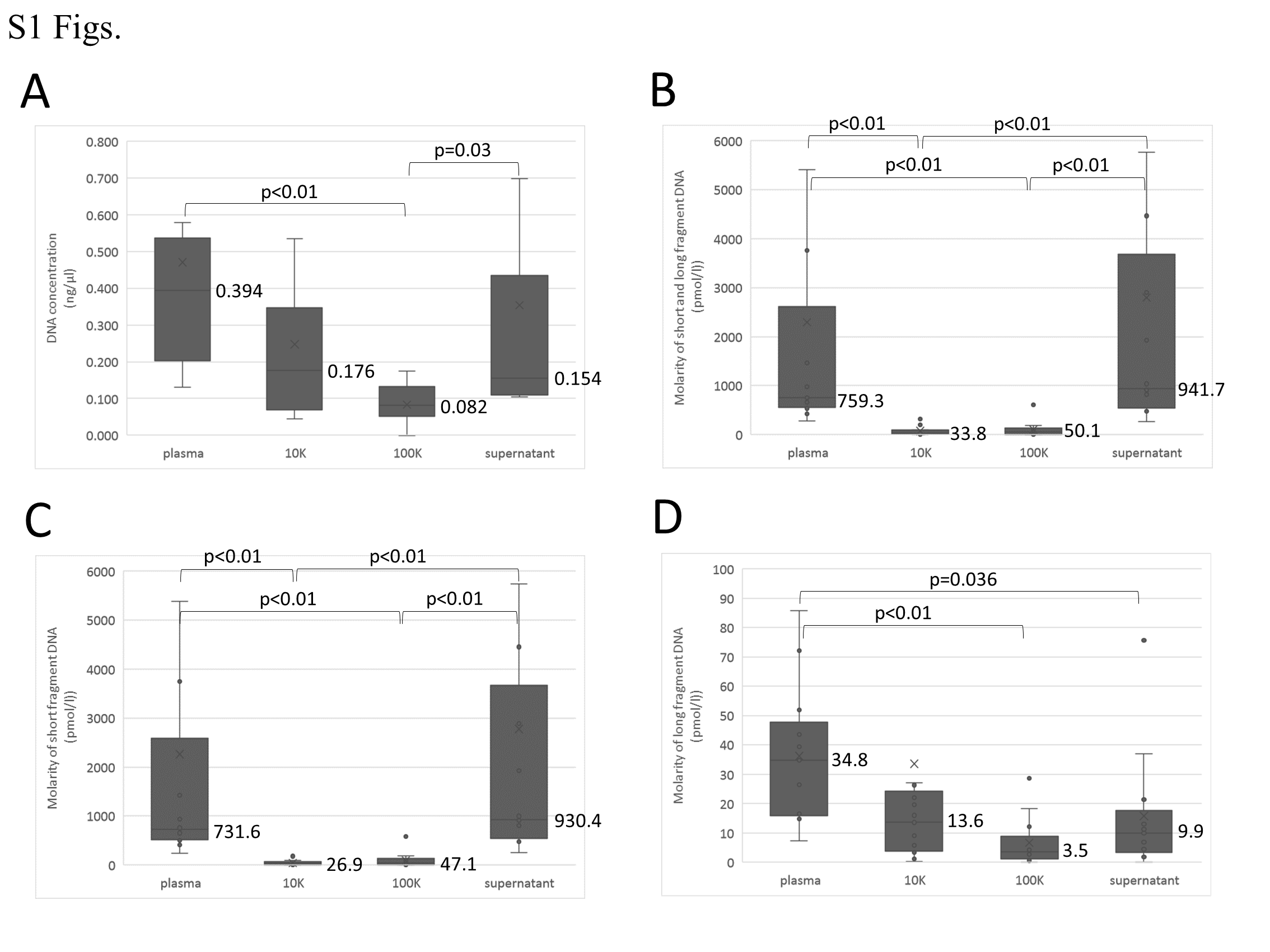

Supplement: S1 Fig — (A-D) Comparison of the DNA concentration, molarity of short fragment DNA, molarity of long fragment DNA, and molarity of combined short and long fragment DNA among plasma, 10K pellets, 100K pellets, and supernatant. (TIF) [file pone.0235611.s001.tif]

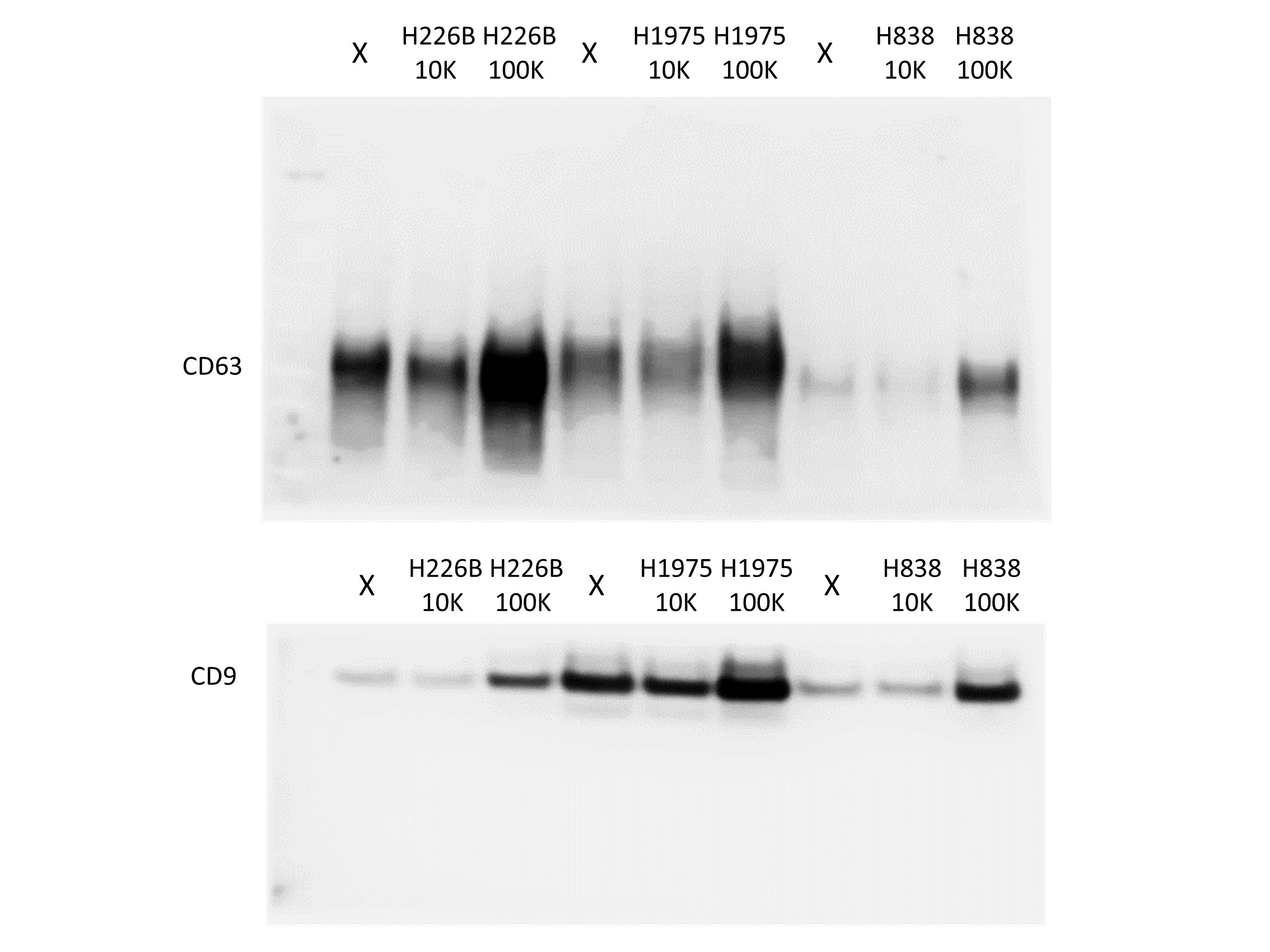

Supplement: S1 Raw image — (TIF) [file pone.0235611.s003.TIF]

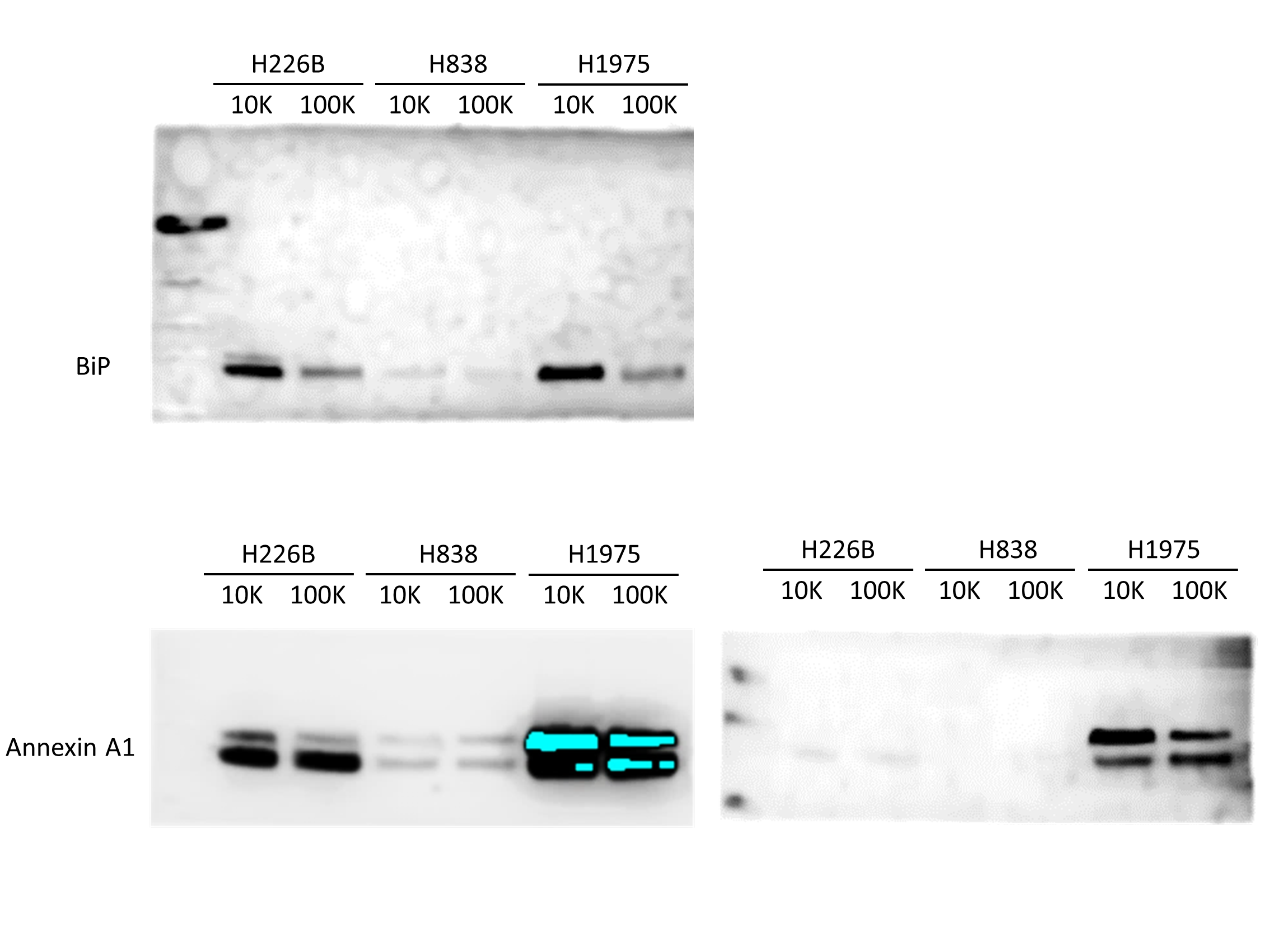

Supplement: S2 Raw image — (TIF) [file pone.0235611.s004.TIF]
