# Supplementary material for: Origin of circulating free DNA in patients with lung cancer
Source: PLoS One. 2020 Jul 7;15(7):e0235611. doi: 10.1371/journal.pone.0235611 (PMC7340299; doi:10.1371/journal.pone.0235611)
Supplement: S1 Table — (DOCX) [file pone.0235611.s002.docx]

**The data of figure 1A**

| case | diagnosis | cfDNA concentration(ng/ml plasma) |
| --- | --- | --- |
| 1 | Lung cancer patients | 18.0 |
| 2 | Lung cancer patients | 175.2 |
| 3 | Lung cancer patients | 17.2 |
| 4 | Lung cancer patients | 35.7 |
| 6 | Lung cancer patients | 11.1 |
| 7 | Lung cancer patients | 8.1 |
| 8 | Lung cancer patients | 14.2 |
| 9 | Lung cancer patients | 10.5 |
| 10 | Lung cancer patients | 10.2 |
| 11 | Lung cancer patients | 29.5 |
| 12 | Lung cancer patients | 37.9 |
| 14 | Lung cancer patients | 17.1 |
| 15 | Lung cancer patients | 31.7 |
| 16 | Lung cancer patients | 8.9 |
| 17 | Lung cancer patients | 41.8 |
| 19 | Lung cancer patients | 9.8 |
| 20 | Lung cancer patients | 9.4 |
| 21 | Lung cancer patients | 7.4 |
| 22 | Lung cancer patients | 10.5 |
| 24 | Lung cancer patients | 25.1 |
| 25 | Lung cancer patients | 21.4 |
| 26 | Lung cancer patients | 11.4 |
| 28 | Lung cancer patients | 6.0 |
| 30 | Lung cancer patients | 17.9 |
| 31 | Lung cancer patients | 9.1 |
| 32 | Lung cancer patients | 15.2 |
| 33 | Lung cancer patients | 31.8 |
| 34 | Lung cancer patients | 11.2 |
| 35 | Lung cancer patients | 13.9 |
| 36 | Benign pulmonary patients | 19.4 |
| 37 | Lung cancer patients | 5.9 |
| 38 | Lung cancer patients | 14.6 |
| 40 | Lung cancer patients | 12.9 |
| 41 | Lung cancer patients | 6.7 |
| 42 | Lung cancer patients | 9.0 |
| 43 | Lung cancer patients | 18.2 |
| 44 | Benign pulmonary patients | 11.1 |
| 46 | Benign pulmonary patients | 7.9 |
| 47 | Lung cancer patients | 13.7 |
| 48 | Lung cancer patients | 7.9 |
| 49 | Lung cancer patients | 7.5 |
| 50 | Lung cancer patients | 4.4 |
| 51 | Lung cancer patients | 13.9 |
| 52 | Lung cancer patients | 23.7 |
| 53 | Benign pulmonary patients | 7.7 |
| 55 | Lung cancer patients | 9.2 |
| 56 | Lung cancer patients | 13.8 |
| 57 | Lung cancer patients | 13.1 |
| 58 | Lung cancer patients | 8.7 |
| 60 | Benign pulmonary patients | 4.3 |
| 61 | Lung cancer patients | 6.1 |
| 62 | Benign pulmonary patients | 6.4 |
| 63 | Lung cancer patients | 7.0 |
| 64 | Lung cancer patients | 10.1 |
| 65 | Lung cancer patients | 4.6 |
| 66 | Benign pulmonary patients | 13.9 |
| 68 | Lung cancer patients | 17.9 |
| 70 | Benign pulmonary patients | 20.1 |
| 71 | Lung cancer patients | 63.2 |
| 72 | Benign pulmonary patients | 12.7 |
| 73 | Lung cancer patients | 9.2 |
| 74 | Benign pulmonary patients | 20.2 |
| 76 | Lung cancer patients | 12.7 |
| 77 | Lung cancer patients | 5.4 |
| 78 | Benign pulmonary patients | 9.6 |
| 79 | Lung cancer patients | 10.5 |
| 80 | Lung cancer patients | 10.1 |
| 81 | Benign pulmonary patients | 8.1 |
| 82 | Lung cancer patients | 10.0 |
| 83 | Lung cancer patients | 7.4 |
| 84 | Lung cancer patients | 14.2 |
| 85 | Lung cancer patients | 13.9 |
| 86 | Benign pulmonary patients | 3.7 |
| 87 | Lung cancer patients | 7.7 |
| 88 | Lung cancer patients | 6.6 |
| 89 | Lung cancer patients | 28.3 |
| 90 | Lung cancer patients | 36.7 |
| 91 | Benign pulmonary patients | 13.0 |
| 92 | Lung cancer patients | 5.7 |
| 93 | Benign pulmonary patients | 6.9 |
| 94 | Lung cancer patients | 4.3 |
| 95 | Lung cancer patients | 4.1 |
| 96 | Lung cancer patients | 7.0 |
| 97 | Lung cancer patients | 10.9 |
| 98 | Lung cancer patients | 3.0 |
| 99 | Lung cancer patients | 4.5 |
| 101 | Lung cancer patients | 5.9 |
| 102 | Lung cancer patients | 7.6 |
| 103 | Lung cancer patients | 4.1 |
| 104 | Benign pulmonary patients | 3.8 |
| 106 | Lung cancer patients | 3.9 |
| 107 | Lung cancer patients | 15.4 |
| 109 | Benign pulmonary patients | 2.9 |
| 110 | Lung cancer patients | 3.6 |
| 111 | Lung cancer patients | 3.1 |
| 112 | Lung cancer patients | 5.2 |
| 115 | Lung cancer patients | 25.6 |
| 116 | Benign pulmonary patients | 2.8 |
| 117 | Lung cancer patients | 8.8 |
| 118 | Lung cancer patients | 6.7 |
| 119 | Lung cancer patients | 6.7 |
| 120 | Lung cancer patients | 17.2 |
| 121 | Lung cancer patients | 8.5 |
| 122 | Lung cancer patients | 2.6 |
| 123 | Lung cancer patients | 4.6 |
| 125 | Lung cancer patients | 12.9 |
| 126 | Lung cancer patients | 3.1 |
| 129 | Lung cancer patients | 8.4 |
| 130 | Lung cancer patients | 2.7 |
| 131 | Lung cancer patients | 10.3 |
| H1 | Healthy individuals | 8.7 |
| H2 | Healthy individuals | 8.0 |
| H3 | Healthy individuals | 9.6 |
| H4 | Healthy individuals | 7.3 |
| H5 | Healthy individuals | 3.1 |
| H6 | Healthy individuals | 22.9 |
| H7 | Healthy individuals | 4.9 |
| H8 | Healthy individuals | 9.4 |
| H9 | Healthy individuals | 3.6 |
| H10 | Healthy individuals | 8.9 |
| H11 | Healthy individuals | 7.1 |
| H12 | Healthy individuals | 3.7 |
| H13 | Healthy individuals | 11.3 |
| H14 | Healthy individuals | 6.9 |
| H15 | Healthy individuals | 11.2 |
| H16 | Healthy individuals | 4.9 |
| H17 | Healthy individuals | 5.4 |
| H18 | Healthy individuals | 7.3 |
| H19 | Healthy individuals | 9.0 |
| H20 | Healthy individuals | 7.7 |

**The data of figure 1B**

| case | stage | cfDNA concentration(ng/ml plasma) |
| --- | --- | --- |
| 6 | Ⅰ | 11.1 |
| 7 | Ⅰ | 8.1 |
| 9 | Ⅰ | 10.5 |
| 10 | Ⅰ | 10.2 |
| 12 | Ⅰ | 37.9 |
| 16 | Ⅰ | 8.9 |
| 19 | Ⅰ | 9.8 |
| 25 | Ⅰ | 21.4 |
| 26 | Ⅰ | 11.4 |
| 28 | Ⅰ | 6.0 |
| 32 | Ⅰ | 15.2 |
| 35 | Ⅰ | 13.9 |
| 42 | Ⅰ | 9.0 |
| 43 | Ⅰ | 18.2 |
| 47 | Ⅰ | 13.7 |
| 48 | Ⅰ | 7.9 |
| 49 | Ⅰ | 7.5 |
| 50 | Ⅰ | 4.4 |
| 57 | Ⅰ | 13.1 |
| 58 | Ⅰ | 8.7 |
| 64 | Ⅰ | 10.1 |
| 65 | Ⅰ | 4.6 |
| 68 | Ⅰ | 17.9 |
| 73 | Ⅰ | 9.2 |
| 76 | Ⅰ | 12.7 |
| 83 | Ⅰ | 7.4 |
| 84 | Ⅰ | 14.2 |
| 87 | Ⅰ | 7.7 |
| 88 | Ⅰ | 6.6 |
| 92 | Ⅰ | 5.7 |
| 94 | Ⅰ | 4.3 |
| 96 | Ⅰ | 7.0 |
| 98 | Ⅰ | 3.0 |
| 99 | Ⅰ | 4.5 |
| 102 | Ⅰ | 7.6 |
| 111 | Ⅰ | 3.1 |
| 115 | Ⅰ | 25.6 |
| 117 | Ⅰ | 8.8 |
| 118 | Ⅰ | 6.7 |
| 119 | Ⅰ | 6.7 |
| 126 | Ⅰ | 3.1 |
| 131 | Ⅰ | 10.3 |
| 8 | Ⅱ | 14.2 |
| 30 | Ⅱ | 17.9 |
| 52 | Ⅱ | 23.7 |
| 61 | Ⅱ | 6.1 |
| 79 | Ⅱ | 10.5 |
| 95 | Ⅱ | 4.1 |
| 101 | Ⅱ | 5.9 |
| 103 | Ⅱ | 4.1 |
| 123 | Ⅱ | 4.6 |
| 129 | Ⅱ | 8.4 |
| 130 | Ⅱ | 2.7 |
| 1 | Ⅲ | 18.0 |
| 15 | Ⅲ | 31.7 |
| 17 | Ⅲ | 41.8 |
| 20 | Ⅲ | 9.4 |
| 22 | Ⅲ | 10.5 |
| 33 | Ⅲ | 31.8 |
| 34 | Ⅲ | 11.2 |
| 41 | Ⅲ | 6.7 |
| 51 | Ⅲ | 13.9 |
| 55 | Ⅲ | 9.2 |
| 63 | Ⅲ | 7.0 |
| 80 | Ⅲ | 10.1 |
| 120 | Ⅲ | 17.2 |
| 121 | Ⅲ | 8.5 |
| 122 | Ⅲ | 2.6 |
| 125 | Ⅲ | 12.9 |
| 2 | Ⅳ | 175.2 |
| 3 | Ⅳ | 17.2 |
| 4 | Ⅳ | 35.7 |
| 11 | Ⅳ | 29.5 |
| 14 | Ⅳ | 17.1 |
| 21 | Ⅳ | 7.4 |
| 24 | Ⅳ | 25.1 |
| 31 | Ⅳ | 9.1 |
| 37 | Ⅳ | 5.9 |
| 38 | Ⅳ | 14.6 |
| 40 | Ⅳ | 12.9 |
| 56 | Ⅳ | 13.8 |
| 71 | Ⅳ | 63.2 |
| 77 | Ⅳ | 5.4 |
| 82 | Ⅳ | 10.0 |
| 85 | Ⅳ | 13.9 |
| 90 | Ⅳ | 36.7 |
| 97 | Ⅳ | 10.9 |
| 106 | Ⅳ | 3.9 |
| 107 | Ⅳ | 15.4 |
| 110 | Ⅳ | 3.6 |
| 112 | Ⅳ | 5.2 |

**The data of figure 1C, 2**

| case | metastasis | cfDNA concentration(ng/ml plasma) | Short(pmol/l) | Long(pmol/l) |
| --- | --- | --- | --- | --- |
| 1 | Absent | 17.958 | 489.8 | 41.3 |
| 2 | Present | 175.2 | 14154.2 | 106.6 |
| 3 | Present | 17.22 | 459 | 24.7 |
| 4 | Present | 35.658 | 1219.6 | 58.6 |
| 6 | Absent | 11.058 | 582.4 | 12 |
| 7 | Absent | 8.118 | 201.4 | 9.2 |
| 8 | Absent | 14.238 | 355.5 | 9.3 |
| 9 | Absent | 10.458 | 624.7 | 3.9 |
| 10 | Absent | 10.218 | 548.3 | 9.9 |
| 11 | Present | 29.46 | 1493.5 | 19.7 |
| 12 | Absent | 37.92 | 674.8 | 64.6 |
| 14 | Present | 17.082 | 303.9 | 54.9 |
| 15 | Absent | 31.68 | 967.6 | 173.3 |
| 16 | Absent | 8.862 | 276.4 | 18.5 |
| 17 | Absent | 41.82 | 4229.4 | 921.3 |
| 19 | Absent | 9.84 | 190.5 | 12.8 |
| 20 | Absent | 9.378 | 703.1 | 0.9 |
| 21 | Present | 7.44 | 1000.7 | 2.9 |
| 22 | Absent | 10.542 | 646.1 | 2.6 |
| 24 | Present | 25.098 | 1974.2 | 9.1 |
| 25 | Absent | 21.36 | 2067.9 | 10.2 |
| 26 | Absent | 11.442 | 219.4 | 17.9 |
| 28 | Absent | 5.97 | 392.9 | 1 |
| 30 | Absent | 17.922 | 6020.9 | 227 |
| 31 | Absent | 9.078 | 7874.9 | 195.6 |
| 32 | Absent | 15.222 | 781.5 | 21.7 |
| 33 | Absent | 31.818 | 1156.4 | 31.9 |
| 34 | Absent | 11.178 | 881.5 | 3.5 |
| 35 | Absent | 13.902 | 1284.3 | 15.3 |
| 37 | Present | 5.88 | 304.7 | 1.2 |
| 38 | Present | 14.562 | 400.8 | 30.4 |
| 40 | Absent | 12.942 | 154.4 | 15.3 |
| 41 | Absent | 6.66 | 440.8 | 0.6 |
| 42 | Absent | 9.042 | 657.2 | 1.4 |
| 43 | Absent | 18.222 | 429.6 | 21.9 |
| 47 | Absent | 13.698 | 462 | 28.3 |
| 48 | Absent | 7.92 | 683.9 | 11 |
| 49 | Absent | 7.542 | 381.4 | 2.7 |
| 50 | Absent | 4.41 | 221.8 | 0.9 |
| 51 | Absent | 13.878 | 843 | 23.9 |
| 52 | Absent | 23.658 | 302.6 | 58 |
| 55 | Absent | 9.18 | 371.6 | 0.9 |
| 56 | Present | 13.842 | 1009.3 | 5.2 |
| 57 | Absent | 13.14 | 498 | 20.3 |
| 58 | Absent | 8.718 | 642.9 | 23.9 |
| 61 | Absent | 6.066 | 431.6 | 9.4 |
| 63 | Absent | 7.002 | 578.7 | 1.7 |
| 64 | Absent | 10.14 | 669.7 | 15 |
| 65 | Absent | 4.608 | 238.3 | 7.6 |
| 68 | Absent | 17.862 | 428.3 | 21.5 |
| 71 | Present | 63.198 | 8862.7 | 23.3 |
| 73 | Absent | 9.162 | 604 | 3.9 |
| 76 | Absent | 12.738 | 3816 | 235.6 |
| 77 | Absent | 5.43 | 441.3 | 2.3 |
| 79 | Absent | 10.518 | 715 | 12.5 |
| 80 | Absent | 10.14 | 648.9 | 6.6 |
| 82 | Present | 9.96 | 401.9 | 41.8 |
| 83 | Absent | 7.422 | 690.3 | 14.3 |
| 84 | Absent | 14.238 | 1387.3 | 10 |
| 85 | Present | 13.878 | 760.2 | 17.7 |
| 87 | Absent | 7.74 | 637.4 | 12 |
| 88 | Absent | 6.6 | 436.2 | 14.1 |
| 90 | Present | 36.72 | 4542.3 | 20.3 |
| 92 | Absent | 5.676 | 523.7 | 7.6 |
| 94 | Absent | 4.308 | 176 | 16 |
| 95 | Absent | 4.074 | 394.4 | 6.8 |
| 96 | Absent | 7.038 | 838.1 | 11.5 |
| 97 | Present | 10.92 | 364.8 | 37.3 |
| 98 | Absent | 2.952 | 137.8 | 11 |
| 99 | Absent | 4.47 | 212.2 | 2 |
| 101 | Absent | 5.88 | 394.9 | 11.4 |
| 102 | Absent | 7.602 | 457 | 5 |
| 103 | Absent | 4.14 | 412.9 | 5.5 |
| 106 | Present | 3.924 | 461.5 | 13.8 |
| 107 | Present | 15.438 | 1249.2 | 15.9 |
| 110 | Present | 3.576 | 205.6 | 11 |
| 111 | Absent | 3.054 | 131.4 | 0 |
| 112 | Absent | 5.172 | 103.4 | 8.6 |
| 115 | Absent | 25.56 | 2123.9 | 33.1 |
| 117 | Absent | 8.838 | 560 | 9.8 |
| 118 | Absent | 6.678 | 225.6 | 11.1 |
| 119 | Absent | 6.702 | 661.8 | 6.4 |
| 120 | Absent | 17.202 | 870 | 31.6 |
| 121 | Absent | 8.46 | 628.4 | 16.2 |
| 122 | Absent | 2.61 | 293 | 6 |
| 123 | Absent | 4.614 | 514.3 | 14.2 |
| 125 | Absent | 12.942 | 876.5 | 1.7 |
| 126 | Absent | 3.096 | 230.8 | 0.8 |
| 129 | Absent | 8.358 | 569.1 | 17.4 |
| 130 | Absent | 2.712 | 202.5 | 5.5 |
| 131 | Absent | 10.338 | 359.6 | 23.5 |

**The data of figure 4**

| case | short(pmol/l) | long(pmol/l) | metastasis |
| --- | --- | --- | --- |
| 1 | 83.7 | 8.4 | Absent |
| 2 | 144.2 | 12.7 | Absent |
| 3 | 114.9 | 28.8 | Present |
| 4 | 1941.5 | 67.3 | Present |
| 5 | 0.0 | 1.2 | Absent |
| 6 | 123.9 | 15.7 | Absent |
| 7 | 1791.1 | 139.9 | Present |
| 8 | 417.7 | 15.1 | Absent |
| 9 | 28.0 | 11.7 | Absent |
| 10 | 210.2 | 5.4 | Present |
| 11 | 3.8 | 1.5 | Absent |
| 12 | 2028.7 | 145.1 | Present |
| 13 | 496.2 | 30.9 | Present |
| 14 | 1010.7 | 6.1 | Present |
| 15 | 1968.6 | 33.6 | Present |
| 16 | 4631.9 | 37.6 | Present |
| 17 | 1956.0 | 26.0 | Absent |
| 18 | 772.6 | 16.7 | Present |
| 19 | 353.0 | 15.8 | Present |
| 20 | 1359.2 | 23.1 | Present |

**The data of figure 5, S1**

| sample | centrifugation | Concentration  (ng/µl) | Short  (pmol/l) | Long  (pmol/l) | short+long  (pmol/l) | L858R  (copies) | allele frequency(%) |
| --- | --- | --- | --- | --- | --- | --- | --- |
| A | plasma | 0.478 | 528.8 | 52 | 580.8 | 1 | 0.25 |
| A | 10K | 0.493 | 33.6 | 288.3 | 321.9 | 0 | 0 |
| A | 100K | 0.000 | 4.1 | 0 | 4.1 | 0 | 0 |
| A | supernatant | 0.104 | 912.1 | 1.8 | 913.9 | 1 | 1.1 |
| B | plasma | 0.245 | 731.6 | 27.7 | 759.3 | 1 | 0.5 |
| B | 10K | 0.176 | 12.6 | 16 | 28.6 | 0 | 0 |
| B | 100K | 0.082 | 29 | 3.3 | 32.3 | 0 | 0 |
| B | supernatant | 0.154 | 807.1 | 9.9 | 817 | 1 | 1 |
| C | plasma | 0.323 | 943.4 | 39.4 | 982.8 | 14 | 6.4 |
| C | 10K | 0.160 | 1.2 | 13.6 | 14.8 | 0 | 0 |
| C | 100K | 0.061 | 0 | 4.3 | 4.3 | 3 | 17 |
| C | supernatant | 0.152 | 930.4 | 11.3 | 941.7 | 16 | 13 |
| D | plasma | 0.448 | 762.1 | 72.2 | 834.3 | 15 | 3.6 |
| D | 10K | 0.069 | 1.1 | 0.2 | 1.3 | 0 | 0 |
| D | 100K | 0.118 | 11.7 | 18.4 | 30.1 | 2 | 2.3 |
| D | supernatant | 0.198 | 1000.3 | 36.9 | 1037.2 | 6 | 4.4 |
| E | plasma | 0.294 | 246.9 | 34.8 | 281.7 | 4 | 1.3 |
| E | 10K | 0.201 | 14.2 | 19.6 | 33.8 | 0 | 0 |
| E |  | 0.146 | 47.1 | 12.1 | 59.2 | 0 | 0 |
| E | supernatant | 0.106 | 258.7 | 7.2 | 265.9 | 1 | 2.6 |
| F | plasma | 0.160 | 654.7 | 16.6 | 671.3 | 13 | 12 |
| F | 10K | 0.049 | 23.3 | 5.7 | 29 | 0 | 0 |
| F | 100K | 0.097 | 75.7 | 5 | 80.7 | 0 | 0 |
| F | supernatant | 0.117 | 475 | 2.3 | 477.3 | 20 | 22 |
| G | plasma | 1.060 | 502.6 | 35.5 | 538.1 | 60 | 6.7 |
| G | 10K | 0.888 | 51.7 | 27.1 | 78.8 | 1 | 0.16 |
| G | 100K | 0.000 | 95.3 | 5.5 | 100.8 | 2 | 5 |
| G | supernatant | 0.227 | 1,929.70 | 0 | 1929.7 | 67 | 23.3 |
| H | plasma | 0.131 | 659.7 | 7.3 | 667 | 14 | 14 |
| H | 10K | 0.067 | 26.9 | 4 | 30.9 | 0 | 0 |
| H | 100K | 0.056 | 1.4 | 0.9 | 2.3 | 1 | 50 |
| H | supernatant | 0.113 | 547.9 | 4.5 | 552.4 | 17 | 14 |
| I | plasma | 0.496 | 3748.6 | 14.8 | 3763.4 | 1 | 0.3 |
| I | 10K | 0.044 | 98.1 | 1.1 | 99.2 | 0 | 0 |
| I | 100K | 0.059 | 176.8 | 2.7 | 179.5 | 0 | 0 |
| I | supernatant | 0.478 | 4452.3 | 13.9 | 4466.2 | 1 | 0.3 |
| J | plasma | 1.357 | 13407.9 | 85.7 | 13493.6 | 138 | 14.7 |
| J | 10K | 0.182 | 180.7 | 22.1 | 202.8 | 2 | 2 |
| J | 100K | 0.174 | 585.4 | 28.6 | 614 | 9 | 12 |
| J | supernatant | 1.760 | 15683.5 | 75.7 | 15759.2 | 216 | 16 |
| K | plasma | 0.394 | 1428.2 | 43.5 | 1471.7 | 28 | 6.3 |
| K | 10K | 0.534 | 59.7 | 26.3 | 86 | 0 | 0 |
| K | 100K | 0.083 | 3.8 | 3.5 | 7.3 | 2 | 12 |
| K | supernatant | 0.392 | 2887.4 | 13.1 | 2900.5 | 42 | 14.3 |
| L | plasma | 0.579 | 5381.2 | 26.4 | 5407.6 | 59 | 11.3 |
| L | 10K | 0.185 | 81.6 | 3.4 | 85 | 1 | 2.3 |
| L | 100K | 0.047 | 186.4 | 0.6 | 187 | 2 | 11 |
| L | supernatant | 0.699 | 5742.6 | 21.4 | 5764 | 37 | 8.4 |
| M | plasma | 0.158 | 410.3 | 15.1 | 425.4 | 11 | 7.6 |
| M | 10K | 0.164 | 1.8 | 9.1 | 10.9 | 2 | 1.3 |
| M | 100K | 0.158 | 48.6 | 1.5 | 50.1 | 0 | 0 |
| M | supernatant | 0.104 | 536.2 | 6.8 | 543 | 17 | 23 |
